# Paracetamol Half-Life Declines over Time After an Acute Overdose

**DOI:** 10.1097/FTD.0000000000001395

**Published:** 2025-09-24

**Authors:** Johannes A. Kroes, Jeroen Poortvliet, Daan J. Touw, Berdien E. Oortgiesen, Marieke G.G. Sturkenboom

**Affiliations:** * University of Groningen, University Medical Center Groningen, Department of Clinical Pharmacy and Pharmacology, Groningen, the Netherlands;; † Frisius Medical Centre, Department of Clinical Pharmacy and Pharmacology, Leeuwarden, the Netherlands;; ‡ University of Groningen, Groningen Research Institute of Pharmacy, Department of Pharmaceutical Analysis, Groningen, the Netherlands; and; § University of Groningen, Groningen Research Institute of Pharmacy, Unit of Pharmacotherapy, -Epidemiology and -Economics, Groningen, the Netherlands.

**Keywords:** paracetamol, half-life, toxicology, overdose, acetylcysteine, emergency medicine

## Abstract

**Background:**

Paracetamol poisoning is one of the most common types of drug-induced poisoning worldwide and can cause severe hepatotoxicity. Acetylcysteine administration is the most effective intervention to prevent hepatotoxicity. Clinically, we observed multiple cases in which the initially prolonged paracetamol half-life decreased during acetylcysteine treatment. This study aimed to investigate the changes in the half-life of paracetamol in patients with paracetamol poisoning treated with acetylcysteine.

**Methods:**

This was a retrospective observational study on patients with paracetamol poisoning who were treated with acetylcysteine. Patients with ≥3 paracetamol plasma concentrations during a single acute overdose were selected from 2 hospitals in the Netherlands. The half-lives of paracetamol in these patients were calculated using consecutive samples. A moderately prolonged paracetamol half-life was defined as a first half-life of >4.0 hours and a severely prolonged paracetamol half-life of >5.5 hours.

**Results:**

Seventy-one paracetamol overdoses from 59 patients were included. The half-life of paracetamol changed from 4.6 (3.5–8.2) h for the first half-life to 2.9 (2.1–3.8) hours for the second half-life (*P* < 0.001). In 20 cases (28%), the initial half-life was moderately prolonged (4.0–5.5 hours), and in 26 cases (37%), it was severely prolonged (>5.5 hours). In all 3 subgroups, the half-life decreased significantly between the first and second half-lives (*P* < 0.001). The reported ingested dose, 4-hour paracetamol concentration, and time to acetylcysteine administration did not differ between the subgroups.

**Conclusions:**

Our results suggest that the half-life of paracetamol decreases during acetylcysteine treatment. Health care professionals should consider repeated measurements of paracetamol concentrations and calculation of half-lives when using paracetamol half-life as a marker of hepatotoxicity, particularly when deciding whether to extend acetylcysteine treatment beyond the duration stated in the current guidelines.

## BACKGROUND

Given the widespread availability of paracetamol, overdoses frequently occur and paracetamol poisoning is one of the most common intentional drug poisonings worldwide.^1–3^ A possible severe outcome of paracetamol poisoning is the development of hepatotoxicity [alanine aminotransferase (ALT) > 1000 IU/L] caused by the toxic metabolite N-acetyl-p-benzoquinone imine (NAPQI). Therapeutic paracetamol is sulfated or glucuronidated to nontoxic metabolites, and only 5%–15% is metabolized to NAPQI, which is immediately conjugated and inactivated by glutathione.^1,4^

At toxic paracetamol doses, the sulfation and glucuronidation pathways are saturated and a higher fraction is metabolized to NAPQI. NAPQI eventually depletes glutathione, leading to hepatotoxicity.^1^ Hepatotoxicity can lead to liver failure, coagulopathy, encephalopathy, renal failure, acidosis, and hypotension.^5^ The most effective intervention for preventing hepatotoxicity is the administration of the antidote acetylcysteine, which prevents the toxic effects of NAPQI in part by replenishing glutathione.

To date, the most reliable parameter for predicting hepatotoxicity is the half-life of paracetamol.^4^ At therapeutic doses, the half-life ranges from 2.0 to 2.5 hours^6,7^ If the half-life is prolonged to more than 4 hours owing to an overdose, the risk of hepatotoxicity increases, and extended treatment with acetylcysteine is required.^4,8,9^ Clinically, we have observed multiple cases in which the initially prolonged half-life of paracetamol decreased during acetylcysteine treatment. This observation may have implications for the duration of acetylcysteine treatment as the risk of toxicity may be lower than initially anticipated.

To determine whether this is indeed a common phenomenon, this study aimed to investigate the changes in the half-life of paracetamol in patients with paracetamol poisoning treated with acetylcysteine.

## METHODS

### Study Design and Setting

This was a retrospective observational study conducted at an academic and top clinical teaching hospital in the Netherlands. The medical ethics committee (Regionale Toetsingscommissie Patiëntgebonden Onderzoek) approved the protocol and waived the requirement for informed consent (MCL Academy 202400361).

### Study Population

The laboratory information system (GLIMS versions 8.11.26 (MCL) and 9.9.6 (UMCG) Clinisys MIPS, Ghent, Belgium) was used as the primary data source. Patients with 3 or more paracetamol plasma concentrations during a single overdose between April 1, 2016, and April 1, 2024 were selected. However, patients with a first sample concentration <75 mg/L were excluded, as they would not have received acetylcysteine if this was a 4-hour postingestion paracetamol concentration, in concordance with Dutch guidelines.^10–12^ Acetylcysteine treatment differed between the 2 hospitals. At the academic hospital, until 2019, the patients were treated with an acetylcysteine loading dose of 150 mg/kg; this was followed by 450 mg/kg in 24 hours^12^ Acetylcysteine treatment was continued after 24 hours if the paracetamol concentration exceeded 10 mg/L. After 2019, the SNAP regimen was used, and patients received a loading dose of 100 mg/kg for 2 hours, followed by 200 mg/kg in 10 hours, after which treatment prolongation was considered based on paracetamol plasma concentrations, INR, and ALT.^8^ At the top clinical teaching hospital, the standard acetylcysteine treatment was a 150 mg/kg loading dose, followed by 150 mg/kg in 24 hours^13^ In patients with an ingested dose >30 g or a 4-hour paracetamol concentration >300 mg/L, maintenance treatment was increased to 450 mg/kg in 24 hours^14^ Acetylcysteine treatment was continued at the maintenance dose after 24 hours if the paracetamol concentration was >5 mg/L. Patients could be included more than once if they had multiple acute overdoses. The half-life of paracetamol was calculated as Ln 2/elimination constant Ke. Ke = −(Ln C2-Ln C1)/(T2-T1), where C1 and C2 represent 2 consecutive paracetamol concentrations and T2-T1 is the time interval between these 2 samples.

### Data Collection and Study Definitions

Available patient characteristics (such as sex, age, and hospital), paracetamol concentrations, and sampling times were extracted from electronic health records (EPIC, Verona, WI, version November 2024). Samples below the lower limit of quantification (LLOQ; 5 mg/L) were interpreted as unmeasurable and were replaced with 0.1 mg/L. This value was selected because of the logarithmic nature of the half-life calculations. The half-life was calculated between each pair of consecutive samples: first and second samples (first half-life), second and third samples (second half-life), third and fourth samples (third half-life), and fourth and fifth samples (fourth half-life), if available. A moderately prolonged paracetamol half-life was defined as a first half-life >4.0 hours, whereas a severely prolonged paracetamol half-life was defined as a first half-life >5.5 hours^7^

### Statistical Analyses

Continuous variables are expressed as medians (interquartile range [IQR]), and categorical variables are expressed as numbers with percentages. Patients were categorized into 3 groups based on their first measured half-life: normal (<4.0 hours), moderately prolonged (4.0–5.5 hours), and severely prolonged (>5.5 hours). Differences between these subgroups were analyzed using the Kruskal–Wallis test for continuous outcomes (eg, change in half-life) and the χ^2^ test for categorical variables. To assess the differences in the calculated paracetamol half-lives over time (within-subject comparisons) in these subgroups, Wilcoxon signed-rank tests were used to compare the first and second half-lives.

*P* < 0.05 was considered significant. IBM SPSS Statistics for Windows, version 28 (IBM, Armonk, NY) was used for data analysis.

## RESULTS

### Patient Characteristics

A total of 4259 paracetamol samples were analyzed between April 1, 2016, and April 1, 2024. Seventy-one cases of paracetamol overdose in 59 patients met the inclusion criteria (235 samples). Eight patients had multiple overdoses (ranging from 2 to 5). In a single overdose, 14 cases had 4 paracetamol concentrations and 4 cases had 5 concentrations. The median (IQR) age of the cases at presentation was 26 (21–49) years and 56 (79%) were female (Table 1). A first prolonged half-life was observed in 46 overdoses (65%). All patients were treated with acetylcysteine, as per the local protocols.

**TABLE 1.** Baseline Characteristics [Median (IQR) Unless Otherwise Specified]

Baseline Characteristic	Cases (N = 71)
Female sex; *n* (%)	56 (79)
Age (years)	26 (21–49)
Length (m)	1.69 (1.65–1.78)
Bodyweight (kg)	65 (55–75)
Body mass index (kg/m^2^)	22.9 (20.3–26.0)
Academic Hospital; *n* (%)	
Academic	54 (76)
Top clinical	17 (24)
Risk factor for paracetamol toxicity; *n* (%)	
None	49 (55)
Unknown	6 (9)
Malnourishment	16 (23)
Alcohol abuse	8 (11)
Infection	1 (1)
Cases with multiple risk factors	1 (1)
Mixed intoxication; *n* (%)	32 (45)
Alcohol concentration at presentation known; *n* (%)	13 (18)
Alcohol concentration at presentation (g/L)	0.2 (0.1–1.7)
Ingested dose known; *n* (%)	67 (94)
Ingested dose (g)	25.0 (16.0–42.5)
Ingested dose per bodyweight (mg/kg)	352 (267–510)
Time to first sample after ingestion (h)	4.0 (3.9–5.1)
First paracetamol concentration (mg/L)	175 (142–248)
First paracetamol concentration at 4 h after ingestion and >200 mg/L; *n* (%)	13 (33)
Half-life (h); *n* (%)	
<4.0	25 (35)
4.0–5.5	20 (28)
>5.5	26 (37)
First ALT >1000 IU/L; *n* (%)	1 (1)
First INR; n	1.1 (1.0–1.3); 12
Estimated glomerular filtration rate at presentation (mL/min/1.73 m^2^)	111 (90–128)
Time to acetylcysteine after ingestion (h)	4.4 (2.6–6.2)
Route of acetylcysteine administration; *n* (%)	
Intravenously	68 (96)
Unknown	3 (4)
Duration of acetylcysteine (h); *n* (%)	
0–12 h	10 (16)
0–24 h	21 (30)
>24 h	29 (41)
Acetylcysteine dose per 24 h (mg/kg)	520 (340–630)
Other therapies; *n* (%)	
None	42 (59)
Activated charcoal	22 (31)
Renal replacement therapy	1 (1)
Combination	5 (7)
Overdose outcomes; *n* (%)	
Discharged	67 (94)
Deceased	3 (4)
Unknown	1 (1)

### Paracetamol Overdoses

**FIGURE 1.**
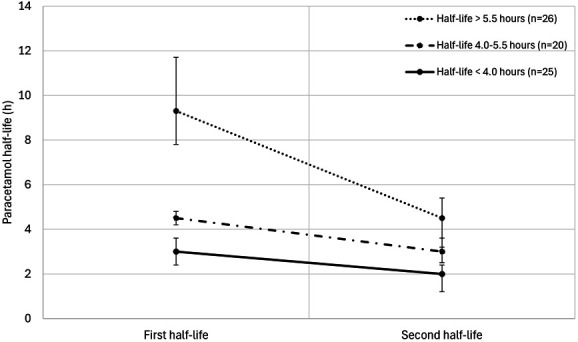
Paracetamol half-lives. Median (IQR) paracetamol half-life change for patients with a normal half-life (<4.0 hours, solid line), a moderately prolonged half-life (4.0–5.5 hours, dash-dotted line), and a severely prolonged half-life (>5.5 hours, dotted line). The third and fourth half-lives are not shown due to the small number of cases. The second half-life was significantly decreased compared with the first half-life in all groups (*P* < 0.001, Wilcoxon signed-rank tests). For the number of patients per group, please refer to Table 2.

Table 2 summarizes the median (IQR) paracetamol concentrations, calculated half-lives, and the corresponding times. The half-life changed from 4.6 (3.5–8.2) h for the first half-life to 2.9 (2.1–3.8) h for the second half-life (*P* < 0.001). In 20 cases (28%), the initial half-life was moderately prolonged (4.0–5.5 hours), and in 26 cases (37%), it was severely prolonged (>5.5 hours). In all 3 subgroups, the half-life decreased significantly between the first and second half-lives (*P* < 0.001 in all subgroups). Between the subgroups, the change in half-life was significantly larger when comparing the severely prolonged half-lives with those of the other subgroups (−4.9 (−8.7 to −2.5) versus −1.8 (−2.0 to −1.1) and −1.1 (−1.9 to −0.4), *P* < 0.001). The course of the paracetamol half-lives for these 3 groups is shown in Figure 1. In 9 (75%) of the 12 cases of a severely prolonged half-life, the (reliable) 4-hour paracetamol concentration was below 200 mg/L.

**TABLE 2.** Paracetamol Samples [Median (IQR)]

Paracetamol Concentration (mg/L)	All Cases		Half-Life >5.5 h		Half-Life 4.0–5.5 h		Half-Life <4.0 h		*P* *
		N		N		N		N	
Sample 1	175 (142–248)	71	185 (143–326)	26	185 (129–242)	20	172 (142–209)	25	0.520
Sample 2	70 (46–103)	71	97 (70–198)	26	64 (43–101)	20	51 (24–75)	25	<0.001
Sample 3	7.7 (0.1–23)	71	26 (13–52)	26	7 (5–15)	20	0.1 (0.1–5)	25	<0.001
Sample 4	6.6 (0.1–18)	18	8.1 (5.3–28)	13	0.1 (0.1–5)	4	6.4	1	0.224
Sample 5	0.1 (0.1–7.0)	4	0.1 (0.1–7.0)	4	—	0	—	0	—
Time (hours)									
Between samples 1-2	5.6 (4.0–9.5)	71	6.8 (4.1–11.0)	26	5.1 (4.0–9.1)	20	4.4 (4.0–7.4)	25	0.195
Between samples 2-3	9.0 (6.4–12.9)	71	8.9 (6.4–11.0)	26	8.5 (6.4–10.4)	20	11.8 (7.0–14.2)	25	0.369
Between samples 3-4	13.3 (6.4–19.3)	18	16.5 (8.3–24.0)	13	7.4 (6.8–12.8)	4	4.2	1	0.203
Between samples 4-5	15.9 (15.3–19.6)	4	15.9 (15.3–19.6)	4	—	0	—	0	—
Half-life (hours)									
First	4.6 (3.5–8.2)	71	9.3 (7.8–11.7)	26	4.5 (4.2–4.8)	20	3.0 (2.4–3.6)	25	<0.001
Second	2.9 (2.1–3.8)	71	4.5 (3.2–5.4)	26	3.0 (2.5–3.6)	20	2.0 (1.2–2.4)	25	<0.001
Third	3.9 (2.3–6.2)	18	6.0 (3.7–6.7)	13	1.7 (0.9–3.1)	4	2.3	1	0.104
Fourth	2.0 (1.9–8.9)	4	2.0 (1.9–8.9)	4	—	0	—	0	—
Change in half-life (hours)									
First–second	−1.9 (−3.5–−1.0)	71	−4.9 (−8.7 to −2.5)†	26	−1.8 (−2.0 to −1.1)†	20	−1.1 (−1.9 to −0.4)†	25	<0.001

* Data for comparison of groups were tested using the Kruskal–Wallis test.

† Wilcoxon signed-rank test was used to compare the first and second half-lives within the group *P* < 0.001.

Subgroup analyses (Table 3) of patients with normal, moderately, and severely prolonged paracetamol half-lives did not show any significant differences between the subgroups. The reported ingested dose, 4-hour paracetamol concentration, and time to acetylcysteine treatment did not differ between subgroups. Hepatotoxicity (ALT >1000 IU/L) and fatal outcome were observed more often in cases with a severely prolonged half-life.

**Table 3.** Subgroup Analyses [Data are Presented as Medians (IQR) Unless Otherwise Specified]

Variable	Half-Life >5.5 h	Half-Life 4.0–5.5 h	Half-Life <4.0 h	*P* *
Female sex; n (%)	18 (69)	15 (75)	23 (92)	0.121†
Age (years)	23 (21–42)	34 (22–57)	26 (20–47)	0.334
Body mass index (kg/m^2^)	21 (19–26)	23 (21–27)	24 (20–26)	0.239
Mixed intoxication; n (%)	10 (39)	12 (60)	10 (40)	0.221†
Ingested dose (g)	28 (18–50)	22 (15–50)	23 (18–28)	0.318
Ingested dose per bodyweight (mg/kg)	463 (300–655)	340 (218–667)	326 (267–417)	0.243
First paracetamol concentration (mg/L)	185 (143–326)	185 (129–242)	172 (142–209)	0.520
First paracetamol concentration at 4 h after ingestion and >200 mg/L; n (%)	3 (25)	4 (40)	6 (33)	0.752†
Time to first concentration after ingestion (h)	4.0 (4.0–5.1)	4.0 (3.8–5.3)	4.0 (3.9–4.5)	0.919
Time to acetylcysteine after ingestion (h)	4.0 (2.3–7.5)	5.4 (3.0–6.5)	4.2 (2.5–6.0)	0.666
Peak ALT >1000 IU/L; n (%)	3 (12)	0 (0)	0 (0)	0.071†
Deceased; n (%)	3 (12)	0 (0)	0 (0)	0.094†

* Numerical data for the comparison of groups were tested using the Kruskal–Wallis test.

† Categorical data were tested using the χ^2^ test.

## DISCUSSION

In this retrospective observational study, we found that the half-life of paracetamol decreased significantly during acetylcysteine treatment. This was more pronounced in patients with an initially prolonged half-life. Our findings suggest that acetylcysteine may be effective in reducing the half-life of paracetamol after an overdose.

This study is unique compared with other studies as we collected data from 2 different hospitals over a period of 8 years. These data reflect current clinical practice and are therefore applicable to other clinical settings. Our study is limited by its observational design, as there is a considerable risk of sampling bias. Patients with a shorter half-life will be sampled less often for a third paracetamol concentration, and therefore, patients with a prolonged half-life may be overrepresented. Second, in cases where the paracetamol concentration was below the LLOQ, we assumed the concentration to be 0.1 mg/L. This may have led to overestimation of the observed effect. However, we feel that other assumptions (eg, half of LLOQ, 2.5 mg/L) might lead to an underestimation of the effect, as it is very likely that the patients will have completely cleared the medication by the time unmeasurable blood concentrations are achieved. Unfortunately, data on other parameters associated with hepatotoxicity are limited to ALT, as INR has rarely been reported. In addition, female patients were overrepresented in our population (79%), which may limit the generalizability of our findings owing to possible sex-related differences in paracetamol metabolism.

To the best of our knowledge, the changes in the half-life of paracetamol during acetylcysteine treatment have not yet been investigated. However, the relationship between the half-life of paracetamol and hepatotoxicity has been studied. Prolongation of the paracetamol elimination half-life in untreated patients has been recognized as a key early predictor of hepatotoxicity.^4,15^ Current guidelines state that acetylcysteine treatment should be extended if the patient shows signs of hepatotoxicity.^8,16^ According to a questionnaire in the Netherlands, in 56 of 67 hospitals (84%), clinicians incorporated prolonged paracetamol half-life in the treatment of paracetamol poisoning.^10^ Our results suggest that before the use of paracetamol half-life as a marker for hepatotoxicity, this marker should be redetermined during acetylcysteine treatment. Furthermore, a prolonged half-life was rarely observed in patients with a 4-h paracetamol concentration <200 mg/L.^4^ Remarkably, we found that in 9 of 12 cases (75%) with a prolonged half-life, the paracetamol concentration 4 hours after ingestion was below 200 mg/L. In addition, the ingested dose, 4-hour paracetamol concentration, and time to acetylcysteine treatment did not differ between the subgroups. This suggests that early concentrations alone may underestimate risk in patients with impaired paracetamol metabolism or elimination. Based on these findings, we recommend repeated half-life calculations whenever an initially prolonged half-life is observed.

It is possible that the initial prolonged half-life was caused by the saturation of the nontoxic sulfation and glucuronidation pathways of paracetamol. The shortening of the half-life of paracetamol might be driven by the treatment with acetylcysteine, as glutathione supplementation might promote the metabolism of paracetamol into nontoxic metabolites.^17^ In addition to restoring depleted glutathione, acetylcysteine is known to stimulate the sulfate conjugation of paracetamol, which might stimulate this nontoxic pathway by functioning as a sulfate donor, leading to a decrease in paracetamol's half-life. Finally, animal studies have suggested that acetylcysteine leads to the reversion of NAPQI to paracetamol.^17–19^ Schiødt et al reported a median paracetamol half-life of 5.4 (range 0.8–120) h in patients treated with acetylcysteine. They observed a median half-life of 3.0 (0.8–10.0) h in patients without hepatotoxicity, 6.4 (1.3–19.0) h in patients with hepatotoxicity, and 18.4 (4.6–120) h in patients with hepatic encephalopathy (which the authors considered as acute liver failure), respectively (*P* < 0.001). Our data contradict these data.^7^ However, in a Danish study, 60% (67/112) of the patients started acetylcysteine more than 8 hours after paracetamol ingestion, whereas in our study, the median time to acetylcysteine treatment was 4.4 hours.

A second clinical implication of our results is that health care professionals should be aware that even in overdoses with a 4-hour paracetamol concentration below 200 mg/L, the half-life of paracetamol can be prolonged, despite similar ingested doses, 4-hour concentrations, and time to initiation of acetylcysteine treatment across subgroups. This finding warrants repeated monitoring of the half-life of paracetamol when initial elimination seems delayed. Future studies should confirm our findings in patients receiving acetylcysteine treatment by analyzing the toxicokinetics of paracetamol and its metabolites.

In our study, hepatotoxicity and fatal outcome were observed more frequently in patients with a severely prolonged half-life. However, caution must be exercised when drawing conclusions from these data due to the small sample size. These findings should be clarified in larger observational studies.

## CONCLUSIONS

Our results suggest that the half-life of paracetamol decreases during acetylcysteine treatment, particularly when its initial half-life was prolonged. Health care professionals should consider repeated measurements of paracetamol concentrations and calculation of half-lives when using paracetamol half-life as a marker of hepatotoxicity, particularly when deciding whether to extend acetylcysteine treatment beyond the duration stated in the current guidelines.
